# Understanding the Mechanisms of Resistance in *EGFR*-Positive NSCLC: From Tissue to Liquid Biopsy to Guide Treatment Strategy

**DOI:** 10.3390/ijms20163951

**Published:** 2019-08-14

**Authors:** Marzia Del Re, Stefania Crucitta, Giulia Gianfilippo, Antonio Passaro, Iacopo Petrini, Giuliana Restante, Angela Michelucci, Stefano Fogli, Filippo de Marinis, Camillo Porta, Antonio Chella, Romano Danesi

**Affiliations:** 1 Unit of Clinical Pharmacology and Pharmacogenetics, Department of Clinical and Experimental Medicine, University of Pisa, 56126 Pisa, Italy; 2 Division of Thoracic Oncology, IEO, European Institute of Oncology, IRCCS, 20141 Milan, Italy; 3 General Pathology, Department of Translational Research & New Technologies in Surgery and Medicine, University of Pisa, 56126 Pisa, Italy; 4 Unit of Molecular Genetics, Department of Laboratory Medicine, University Hospital, 56126 Pisa, Italy; 5 Department of Internal Medicine, University of Pavia, 27100 Pavia, Italy; 6 Division of Translational Oncology, I.R.C.C.S. Istituti Clinici Scientifici Maugeri, 27100 Pavia, Italy; 7 Unit of Respiratory Medicine, Department of Critical Area and Surgical, Medical and Molecular Pathology, University Hospital, 56126 Pisa, Italy

**Keywords:** liquid biopsy, epidermal growth factor receptor, non-Small Cell Lung Cancer, circulating cell-free DNA, tyrosine kinase inhibitors, mechanisms of resistance

## Abstract

Liquid biopsy has emerged as an alternative source of nucleic acids for the management of Epidermal Growth Factor Receptor (*EGFR*)-mutant non-Small Cell Lung Cancer (NSCLC). The use of circulating cell-free DNA (cfDNA) has been recently introduced in clinical practice, resulting in the improvement of the identification of druggable *EGFR* mutations for the diagnosis and monitoring of response to targeted therapy. *EGFR*-dependent (T790M and C797S mutations) and independent (Mesenchymal Epithelial Transition [*MET*] gene amplification, Kirsten Rat Sarcoma [*KRAS*], Phosphatidyl-Inositol 4,5-bisphosphate 3-Kinase Catalytic subunit Alpha isoform [*PI3KCA*], and RAF murine sarcoma viral oncogene homolog B1 [*BRAF*] gene mutations) mechanisms of resistance to EGFR tyrosine kinase inhibitors (TKIs) have been evaluated in plasma samples from NSCLC patients using highly sensitive methods (i.e., digital droplet PCR, Next Generation Sequencing), allowing for the switch to other therapies. Therefore, liquid biopsy is a non-invasive method able to detect the molecular dynamic changes that occur under the pressure of treatment, and to capture tumor heterogeneity more efficiently than is allowed by tissue biopsy. This review addresses how liquid biopsy may be used to guide the choice of treatment strategy in *EGFR*-mutant NSCLC.

## 1. Introduction

The *Epidermal Growth Factor Receptor* mutant (*EGFR^mut^*) is an important molecular subtype of non-small cell lung cancer (NSCLC) and is highly sensitive to anti-EGFR tyrosine kinase inhibitors (TKIs). The *EGFR^mut^* NSCLC is a good model of the “oncogene addiction” theory, in which a specific oncogenic signaling pathway drives the transformation and proliferation of cancer cells [1,2,3]. The identification of the *EGFR* mutations and the related targeted agents allowed an important paradigm shift in the treatment and prognosis of patients with NSCLC harboring these alterations [4,5,6]. At present, several EGFR TKIs are approved for the treatment of NSCLC carrying activating *EGFR^mut^*.

In particular, three different generations of EGFR TKIs are available and have been approved: the first-generation gefitinib and erlotinib; the second-generation afatinib and dacomitinib; and the third-generation osimertinib. The use of EGFR TKIs significantly improved the clinical outcome, i.e., progression-free survival (PFS) and overall response rate (ORR), when compared with standard platinum-based chemotherapy [7,8,9,10,11]. However, the major challenge now is to overcome primary or acquired resistance in NSCLC patients treated with targeted therapy [12]. In fact, even though treatment with EGFR TKIs allows for a durable response, the majority of patients develop progressive disease (PD) after 10–12 months of treatment. In addition, acquired resistance arises and restricts the long-term efficacy of these EGFR TKIs [12].

Since the initial therapeutic choice depends on the genetic identification of individual tumor profiles, tissue biopsy is the gold standard for molecular analysis [13,14]. Nowadays, the introduction into clinical practice of the minimally invasive liquid biopsy, i.e., the analysis of circulating cell-free DNA (cfDNA), allows for a better management of NSCLC patients and the optimization of their therapy [15], especially for the early identification of the increasing number of resistance mutations that may arise during treatment. Several studies have already highlighted the importance of liquid biopsy to detect molecular alterations responsible for the resistance mechanism [16,17]. Dai et al. conducted a study in which the choice of the targeted therapy was made on the basis of molecular analysis of tissue and liquid biopsy; the authors demonstrated a high consistency in *EGFR^mut^* status between plasma and tissue, supporting the use of liquid biopsy to select patients for TKI therapy [18].

Moreover, in a retrospective study, blood samples were collected from 1138 advanced NSCLC patients at presentation and during the progression of the disease. The authors detected sensitizing *EGFR^mut^* in cfDNA of 113 patients, showing a difference between plasma and serum samples. Specifically, the *EGFR^mut^* was detected in cfDNA isolated from plasma of 31 patients, and the *EGFR^mut^* was detected in cfDNA isolated from serum of only 11 patients [19]. Therefore, even though plasma is considered to be a better source of ctDNA for molecular analysis, the results of this study highlight the need to increase our capability to detect druggable mutations by testing serum when plasma is negative.

In a recent study, actionable genomic alterations were analyzed on cfDNA of 116 NSCLC patients due to the lack of tissue samples or a negative molecular tissue analysis. A treatment decision was established in 23% of patients before the first-line therapy and was changed in 32% of patients who progressed to EGFR TKIs, demonstrating that an analysis of cfDNA by Next Generation Sequencing (NGS) improves genetic profiling of advanced NSCLC patients and the use of targeted therapy [20].

The focus of this article is to show how and when liquid biopsy may be used in the choice of treatment strategy in *EGFR*-mutant NSCLC.

## 2. Mechanisms of Resistance to TKIs in *EGFR^mut^* NSCLC and Treatment Strategies

### 2.1. Liquid Biopsy to Track EGFR-Dependent Mechanisms of Primary and Acquired Resistance

The first use of liquid biopsy is to discover the appearance of new point mutations and, since the most commonly acquired resistance mechanism to first/second-generation TKIs is the expansion of clones bearing the T790M mutation in the *EGFR* exon 20, a liquid biopsy can satisfactorily meet this need [21,22]. Due to its steric hindrance, T790M confers resistance to gefitinib, erlotinib, and afatinib, and its detection allows for the use of the third-generation EGFR-TKI osimertinib as a second-line therapy [23]. Several studies have investigated the feasibility of plasma genotyping using digital droplet PCR (ddPCR) or NGS platforms to select patients who progressed during first-line EGFR-TKIs therapy for treatment with osimertinib, demonstrating an overall objective response rate of 70–75% with plasma analysis [24,25,26]. In this context, the phase II APPLE Trial (AZD9291 Treatment on Positive PLasma T790M in EGFR-mutant NSCLC Patients; NCT02856893), a study evaluating osimertinib treatment in T790M positive plasma *EGFR^mut^* NSCLC patients, might provide additional data on this issue [27].

Interestingly, the disappearance of *EGFR* mutations such as T790M or the LREAT747del/T790M double mutant clone at progression to osimertinib has been demonstrated, suggesting the loss of the drug target as a mechanism of resistance [28]. Similarly, an association has been demonstrated between the loss of the T790M mutation and a shorter time to treatment discontinuation (6.1 vs. 15.2 months), suggesting the emergence of pre-existing resistant clones and a range of competing resistance mechanisms [29]. Recently, the *EGFR* C797S mutation has been reported to be an acquired mechanism of resistance to osimertinib [30]. Moreover, it has been reported that if C797S and T790M mutations are detected in *trans*, a combination of first- and third-generation EGFR TKIs may be more effective [31,32].

On the contrary, if mutations are in *cis*, the EGFR TKIs alone or in combination are not able to suppress the EGFR activity. In addition, when osimertinib is administered in a first-line setting and the C797S mutation develops without the presence of the T790M mutation, a re-challenge with first-generation TKIs has been proposed as an effective strategy [33]. To overcome the resistance caused by the C797S mutation, the efficacy of brigatinib in combination with osimertinib has been demonstrated in an in vitro study [34]. In the last few years, a number of studies have been published that use cfDNA to identify the presence of the T790M/C797S mutations or to monitor their amount in order to predict tumor response to treatment [29,35,36]. Overall, considering the use of different platforms (e.g., ddPCR, Real Time PCR, NGS), liquid biopsy has demonstrated a good sensitivity and specificity (>80%) in both the discovery of acquired mutations and monitoring tumor dynamics [37,38].

### 2.2. Using Liquid Biopsy to Track EGFR-Independent Mechanisms of Primary and Acquired Resistance

Considering the *EGFR*-independent mechanism of resistance, multiple primary or secondary mutations have been reported in the following genes: Kirsten rat sarcoma (*KRAS*), phosphatidylinositol 4,5-bisphosphate 3-kinase catalytic subunit alpha isoform (*PI3KCA*), human epidermal growth factor receptor 2 (*HER2*), and RAF murine sarcoma viral oncogene homolog B1 (*BRAF*) [39,40,41]. It is well-known that the occurrence of mutations in the *RAS* gene promotes cell proliferation and drug resistance, by-passing the blockade of EGFR signaling [39]. *KRAS*, a member of the *RAS* family, is highly mutated in NSCLC (15–30%) and is associated with a lack of response to EGFR inhibitors [42]. A recent study investigated the role of concomitant driver mutations (e.g., *KRAS*, *NRAS*, *BRAF*, *PIK3CA*) on the outcome of 133 *EGFR^mut^* NSCLC patients treated with EGFR TKIs [43]. In particular, PFS was significantly shorter in patients with concomitant driver mutations than it was in patients with *EGFR^mut^* only (7 vs. 11.3 months; *p* = 0.04), suggesting that patients with clonal *EGFR* and other sub-clonal driver mutations benefit less from treatment with EGFR TKIs [43].

Interestingly, in cases carrying both *EGFR* and *KRAS* mutations, patients with a *KRAS* allele fraction that was higher than that of the *EGFR^mut^* had a significantly shorter PFS (2.42 vs. 11.09 months; *p* = 0.0081) and a lower response rate (16.7 vs. 57.1%) [43]. Similarly, a study on cfDNA showed that 48.5% of plasma samples were positive for *KRAS* mutation after progression to EGFR TKIs and 39.4% of those had a *KRAS* and *EGFR* co-mutation [44]. Many other studies, on both plasma and tissue samples, detected *KRAS* mutations in *EGFR^mut^* patients with EGFR-TKI resistance, confirming its predictive role in resistance to treatment [29,45,46]. *NRAS* mutations are detected in a low number of NSCLC patients (∼1%), and Q61K, E63K, G12V, and G12R mutations were found in patients with acquired resistance to EGFR TKIs in pre-clinical and clinical studies as a mechanism of resistance to first- and third-generation EGFR TKIs [29,47,48,49,50].

*PI3KCA* mutations may coexist with the *EGFR^mut^* and play a role in resistance to TKIs [51,52]. *BRAF* mutation in NSCLC refractory to EGFR TKIs occurs in ~5–7% of NSCLC patients [53,54], and V600E and G469A mutations seem to co-exist with *EGFR* T790M, mediating acquired resistance in 1% and 10% of patients who progressed to first-generation EGFR-TKIs or osimertinib, respectively [53,55]. Interestingly, a case report showed that a patient positive for *BRAF* V600E mutation at progression to osimertinib benefitted from the combination of a BRAF inhibitor (encorafenib) and osimertinib [56].

Other well-known mechanisms of resistance include the dysregulation of Mesenchymal Epithelial Transition (*MET*) gene signaling, which is involved in the control of cell differentiation [57], proliferation [58,59], and angiogenesis [60,61]. Of note, high *MET* expression or amplification are connected with poor outcomes in patients with NSCLC [62,63]. The *MET* signaling pathway is linked to the EGFR network through the *PI3K/Protein kinase B* (*Akt*) and Mitogen-activated Protein Kinases (*MAPK*) nodes, showing mutual compensation [64]. For these reasons, *MET* activation is one of the potential mechanisms of resistance to EGFR TKIs in NSCLC. As a matter of fact, *MET* amplification is frequently reported as a mechanism of loss of efficacy of EGFR TKI therapy among *EGFR^mut^* patients [65,66,67].

Several studies report the presence of *MET* amplification in NSCLC treated with anti-EGFR TKIs, with or without the T790M mutation [67,68]. A study of 34 NSCLC patients evaluated the amount of *EGFR* mutation in cfDNA as a marker of response/resistance to osimertinib. Eight patients showed early progression during treatment, and a tumor re-biopsy revealed the presence of *MET* amplification in one case [69]. Similarly, the FLAURA trial (AZD9291 Versus Gefitinib or Erlotinib in Patients With Locally Advanced or Metastatic Non-small Cell Lung Cancer, NCT02296125) showed *MET* amplification and *BRAF* mutation as mechanisms of resistance in patients treated with osimertinib, opening up the possibility of future combinations to overcome resistance [70].

A study showed a plasma tissue correlation using NGS in 13 NSCLC patients with acquired resistance to osimertinib. Four patients were found to be positive for *MET* amplification in both plasma and tissue samples. In addition, a survival analysis showed a better PFS/overall survival (OS) in patients without *MET* alterations, confirming *MET* to be a mechanism of resistance to third-generation EGFR TKIs. To overcome this resistance, an exploratory evaluation with a treatment combination of first/third-generation EGFR TKIs and the *MET* inhibitor crizotinib was conducted; partial responses were clinically and radiographically achieved, and a cfDNA analysis was negative for common cancer-related mutations, suggesting the efficacy of the treatment combination [71]. Similarly, a combination of full-dose osimertinib and crizotinib was administered to two patients with emergent *MET* amplification in a liquid biopsy after progression to erlotinib [72,73]. A partial response was achieved without experiencing serious adverse events in one patient [72], while a dose-reduction of crizotinib due to hematological toxicity was needed in the other patient [73].

These cases demonstrate that combination therapy with osimertinib and crizotinib can be effective in patients with *EGFR^mut^* and *MET* amplification detected by liquid biopsy [72,73]. Moreover, cfDNA may help us to understand the molecular response to pharmacological treatment and provide information on clonal heterogeneity, showing the correlation between dynamic changes in the *EGFR* activating mutation (L858R) and *MET* amplification in treatment response [74]. In the last update of the Phase III AURA3 trial (AZD9291 Versus Platinum-Based Doublet-Chemotherapy in Locally Advanced or Metastatic Non-Small Cell Lung Cancer, NCT02151981), an analysis of the ctDNA genomic profile was also carried out in patients with the T790M mutation who progressed on osimertinib during the study, and several resistance mechanisms were observed, including *MET* amplification [75]. In order to overcome resistance, several agents have been developed to target *MET* or its ligand Hepatocyte Growth Factor (*HGF)* [66,72,76,77,78,79], such as small molecules (e.g., capmatinib, tepotinib, and tivantinib) [80,81] or monoclonal antibodies (e.g., onartuzumab and emibetuzumab) [82] and anti-HGF antibodies (e.g., ficlatuzumab and rilotumumab) [83,84]. Moreover, several *MET* inhibitors have been investigated in combination with EGFR TKIs or cytotoxic agents in NSCLC patients who acquired resistance to TKIs due to the appearance of *MET* amplification [66,85,86].

### 2.3. Small-Cell Lung Cancer (SCLC) Transformation: Still a Challenge for Liquid Biopsy?

Transformation to SCLC is reported to be one of the mechanisms of resistance to treatment and has been observed after both first- and subsequent generation of EGFR TKIs [87,88], occurring in approximately 5–14% of patient biopsies at the time of TKI resistance [21,89]. Tumor heterogeneity in *EGFR^mut^* NSCLC has been widely described, and concurrent SCLC transformation and *EGFR* T790M mutation have been reported [90,91,92]. Clinical cases of *EGFR^mut^* NSCLC patients who received TKIs and developed SCLC transformation at progression have been published [91,93]. In one case, SCLC developed after treatment with gefitinib; cisplatin and etoposide were used as a second-line therapy, followed by chemotherapy and immunotherapy with amrubicin, irinotecan, and nivolumab. At this point, the primary lesion that had transformed into SCLC reconverted into an adenocarcinoma with *EGFR* L858R and T790M mutations. Thus, the patient was treated with osimertinib, showing a clinical remission [93].

Similarly, another study presented an *EGFR^mut^* lung adenocarcinoma, which was treated with erlotinib and chemotherapy and was later found to have transformed into SCLC. The patient was treated with cisplatin and irinotecan and then developed resistance to the therapy; the cfDNA revealed the presence of the *EGFR* T790M mutation, allowing for treatment with osimertinib, which resulted in a good clinical response [94].

Two case reports described the transformation into SCLC as a possible mechanism of resistance to afatinib. The first report described a case of a lung adenocarcinoma harboring *EGFR* exon 19 deletion that, after seven months of treatment with afatinib, progressed and showed SCLC transformation at re-biopsy with concomitant *EGFR* exon 19 deletion. Afatinib was discontinued and chemotherapy was administered with a cisplatin and irinotecan regimen, with no disease progression after four cycles of chemotherapy [95]. Similarly, the second case report described a switch of tumor histotype to SCLC with features of a G3 neuroendocrine carcinoma and positivity for exon 19 deletion of *EGFR*. Interestingly, the switch occurred during hepatic progression, which was the only site not responsive to afatinib. Thus, the patient was treated with a carboplatin plus etoposide chemotherapy, showing a complete response [96]. Transformation into SCLC has also been described as a mechanism of acquired resistance to osimertinib [97,98]. Two case reports showed that the transformation into SCLC occurred after 13–18 months of treatment with osimertinib, and a molecular analysis showed the presence of the EGFR^mut^ exon 19 deletion and L858R without the T790M mutation. Patients were treated with a chemotherapy regimen, showing a complete or partial response [97,98].

Despite the fact that transformation into SCLC is difficult to detect by liquid biopsy, a recent publication demonstrated that ctDNA may be analyzed in terms of changes in global copy number to monitor its dynamics in patients with a histological transformation into SCLC. In particular, *TP53* mutation levels change in accordance with the clinical status of the patient and their response to chemotherapy. Moreover, copy number alterations in the avian myelocytomatosis viral oncogene lung carcinoma derived homolog 1 [*MYCL1*], Sry-related HMG box 2 [*SOX2*], and *SOX4* genes and a gain/loss of cancer genes were associated with transformation into SCLC and were linked to genomic instability due to *TP53* mutant clones [99].

## 3. The Clinical Utility of a Liquid Biopsy in Guiding Treatment with EGFR TKIs

The development of sensitive molecular diagnostic tests has increased our knowledge of the genomic landscape of NSCLC, which shows a complex pattern of molecular abnormalities [100]. While in *EGFR^mut^* NSCLC both tumor growth and response to therapies are driven by *EGFR* signaling, the co-occurrence of genomic alterations has been described and used to identify several biological subsets of NSCLC patients with different outcomes to TKI treatments [101]. While, on the one hand, TKIs are effective against cell clones harboring *EGFR*-activating mutations, on the other hand the drug treatment is able to select cell clones carrying different molecular subtypes that are often resistant to TKI treatment [102]. In fact, tumor heterogeneity promotes different mechanisms of resistance at multiple metastatic sites [103,104].

**Figure 1:**
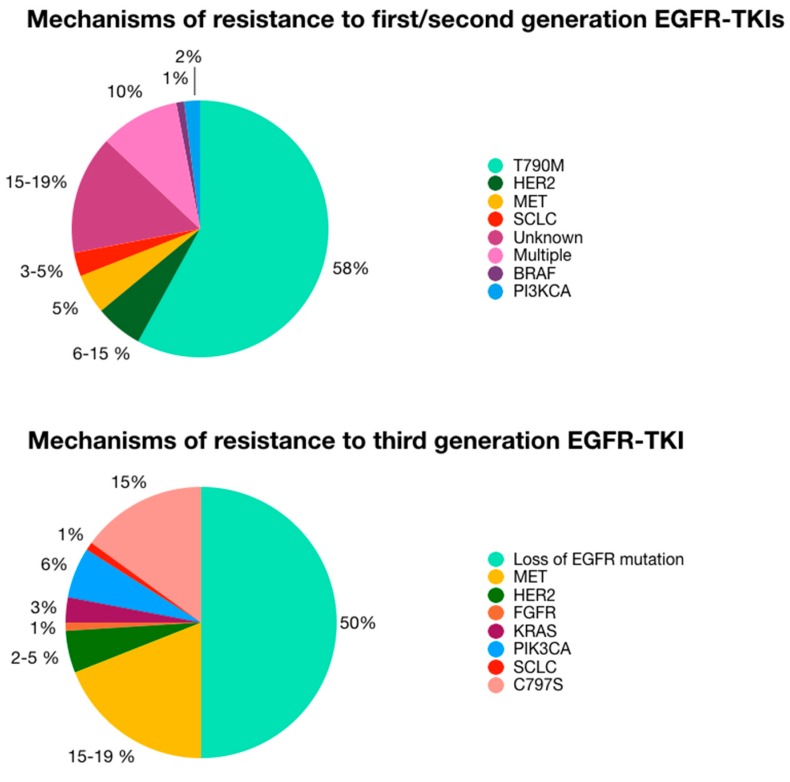
Mechanisms of resistance to EGFR-TKIs and their frequencies. Human Epidermal Growth Factor Receptor 2 (*HER2*), Mesenchymal Epithelial Transition [*MET*], Small Cell Lung Cancer (SCLC), RAF murine sarcoma viral oncogene homolog B1 (*BRAF*), Phosphatidyl-Inositol 4,5-bisphosphate 3-Kinase Catalytic subunit Alpha (*PI3KCA*), Kirsten rat sarcoma (*KRAS*), Fibroblast Growth Factor Receptor (*FGFR*).

Loss of sensitivity to EGFR TKIs may be divided into three groups, based on the mechanisms of selection by treatment: 1) mutations acquired by the target (EGFR), e.g., the T790M or C797S mutations, which reduce the activity of the drug because of a steric hindrance between the target and the drug without diminishing the kinase activity of the receptor [105,106]; 2) activation of a bypass signaling pathway, e.g., *RAS* mutations or *MET* amplification, in the presence of the *EGFR^mut^* [43,69]; and 3) histologic transformation to SCLC [107] (Figure 1A,B). In this context, the ideal approach to monitor tumor dynamics and comprehensively understanding NSCLC’s heterogeneity would be a non-invasive one that is able to capture the molecular events that occur at different tumor sites. For these reasons, liquid biopsy is a useful instrument for following tumor dynamics and heterogeneity [108].

Based on the evidence from the AURA trial, in which an analysis of cfDNA was demonstrated to be comparable to a tissue biopsy for the identification of *EGFR* mutational events, many other studies investigated the predictive potential of liquid biopsy and its advantages for the longitudinal monitoring of tumors, providing results with great relevance to the clinical setting [69,109,110,111,112,113,114,115]. A liquid biopsy, obtained from a routine blood draw of 6–20 mL, can overcome most of the limitations of a tissue biopsy, such as its invasive nature and its inability to represent the tumor’s heterogeneity [116,117,118,119].

Although a liquid biopsy includes an analysis of the circulating free and tumor nucleic acids (DNA and RNA), exosomes, and circulating tumor cells (CTCs) in body fluids, only the analysis of *EGFR^mut^* in cfDNA has been approved for NSCLC patients [15]. Due to the low amount of DNA that can be obtained from CTCs [120], it seems that the major role of CTCs may be that of a prognostic biomarker both in lung cancer and in other solid tumors [121]. However, the lack of Federal Drug Administration (FDA) approval and the cost of CTC isolation remain major issues. Exosomes are micro-vesicles released by cells and contain a wide variety of molecules, such as DNA, RNA, proteins, and lipids, and seem to be implicated in intercellular communication and tumor–host interactions [122]. Exosomes have been described in the literature as markers for the monitoring of tumor dynamics; however, despite their importance, methods to detect and analyze exosomes require further development before they can be introduced into clinical practice [123,124,125,126,127,128].

cfDNA has clearly demonstrated advantages over other markers that make it appropriate for use in clinical practice as a predictive biomarker to monitor response to treatment. In particular, it provides a minimally invasive approach to the early detection of disease recurrence and information about the molecular profile underpinning drug resistance [35]. However, the use of cfDNA presents some limitations and challenges, especially considering the occurrence of false-negative results depending on the following reasons: 1) shedding of cfDNA differs among different tumor types [120]; 2) detection of cfDNA depends on tumor location and volume [129]; 3) detection of cfDNA is lower in patients without progression or who are responding to therapy [130]; and 4) cfDNA shedding is variable [111,131,132].

False-negative results are related to the abovementioned factors; however, the technical limits of detection may also define the level of “false-negatives”, being strictly related to the analytical platform. While it is quite difficult to obtain a technical false-negative result when using very sensitive techniques, such as ddPCR or NGS (which have a lower limit of detection that ranges from 0.001% to 1%) [133,134], technical false-negative results may occur more frequently with less-sensitive techniques, such as the Scorpion Amplified-Refractory Mutation System (SARMS) or Peptide Nucleic Acid-Locked Nucleic Acid (PNA-LNA PCR clamp) [135].

On the other hand, the detection of somatic mutations in cfDNA released from non-cancer cells should be taken into consideration. It is known that clonal hematopoiesis of an indeterminate potential (CHIP) can lead to the development of such mutations [136,137]. Finally, in order to use liquid biopsy in an overall diagnostic/therapeutic strategy for *EGFR^mut^* lung cancers, the analysis of cfDNA should be able to identify *EGFR*-dependent and independent mechanisms of resistance to help us understand the relationship between baseline and resistance mutations.

**Figure 2:**
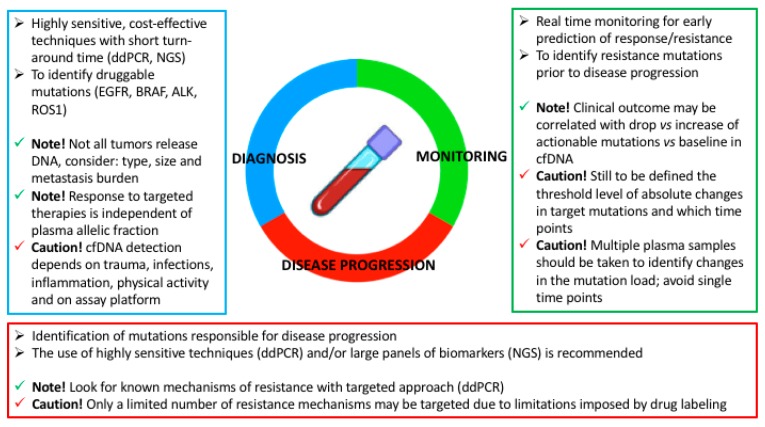
The use of liquid biopsy and circulating cell-free DNA (cfDNA) in clinical practice to guide the choice of treatment strategy. Digital droplet PCR (ddPCR), next generation sequencing (NGS), Epidermal Growth factor Receptor (EGFR), Anaplastic Lymphoma Kinase (ALK), avian UR2 sarcoma virus oncogene homolog 1 ROS1.

A liquid biopsy holds the promise to help us understand the biology and heterogeneity of the tumor and the characteristics of drug-tolerant cells, and has the considerable advantage of short laboratory turn-around time, low cost, mini-invasiveness and the potential of being repeated over time. Such a test may help directing targeted therapies against EGFR and other druggable mutations to prevent the emergence of resistant clones. Figure 2 describes the potential applications of cfDNA in diagnosis, monitoring response and progression of disease and provides suggestions on how a liquid biopsy may be implemented in clinical practice.

In conclusion, a liquid biopsy may be implemented in the clinical management of patients at diagnosis, during treatment (repeated serial liquid biopsies may identify mechanisms of resistance even after transformation into SCLC [93,94]), or during disease progression in order to select an appropriate treatment according to the therapy-dependent clonal selection.
